# Discovery of the cyclotide caripe 11 as a ligand of the cholecystokinin-2 receptor

**DOI:** 10.1038/s41598-022-13142-z

**Published:** 2022-06-02

**Authors:** Mohammad Sadegh Taghizadeh, Bernhard Retzl, Edin Muratspahić, Christoph Trenk, Emilio Casanova, Ali Moghadam, Alireza Afsharifar, Ali Niazi, Christian W. Gruber

**Affiliations:** 1 Center for Physiology and Pharmacology, Medical University of Vienna, 1090 Vienna, Austria; 2 Institute of Biotechnology, Shiraz University, Shiraz, Iran; 3 Center of Plant Virology Research, Shiraz University, Shiraz, Iran

**Keywords:** Peptides, Pharmacology

## Abstract

The cholecystokinin-2 receptor (CCK_2_R) is a G protein-coupled receptor (GPCR) that is expressed in peripheral tissues and the central nervous system and constitutes a promising target for drug development in several diseases, such as gastrointestinal cancer. The search for ligands of this receptor over the past years mainly resulted in the discovery of a set of distinct synthetic small molecule chemicals. Here, we carried out a pharmacological screening of cyclotide-containing plant extracts using HEK293 cells transiently-expressing mouse CCK_2_R, and inositol phosphate (IP1) production as a readout. Our data demonstrated that cyclotide-enriched plant extracts from *Oldenlandia affinis*, *Viola tricolor* and *Carapichea ipecacuanha* activate the CCK_2_R as measured by the production of IP1. These findings prompted the isolation of a representative cyclotide, namely caripe 11 from *C. ipecacuanha* for detailed pharmacological analysis. Caripe 11 is a partial agonist of the CCK_2_R (E_max_ = 71%) with a moderate potency of 8.5 µM, in comparison to the endogenous full agonist cholecystokinin-8 (CCK-8; EC_50_ = 11.5 nM). The partial agonism of caripe 11 is further characterized by an increase on basal activity (at low concentrations) and a dextral-shift of the potency of CCK-8 (at higher concentrations) following its co-incubation with the cyclotide. Therefore, cyclotides such as caripe 11 may be explored in the future for the design and development of cyclotide-based ligands or imaging probes targeting the CCK_2_R and related peptide GPCRs.

## Introduction

The use of plants has attracted wide interest in drug discovery due to the existence of many different and chemically diverse bioactive molecules^1^. Natural products play a significant role in the search for novel cures of cancer, cardiovascular diseases, infectious diseases, and immune disorders^1^. One class of natural products, namely ribosomally synthesized and post-translationally modified peptides (RiPP) are increasingly being recognized for their potential as drug lead molecules or chemical probes^2,3^. They are produced in bacteria, archaea, and eukaryotes and since they are true gene products, their biosynthetic precursor sequences are accessible via mining of genomes and transcriptomes.

Their extensive structural variety combined with restricted conformational flexibility—frequently achieved by disulfide bond formation and cyclization—is considered to lead to improved affinity and selectivity for target proteins as well as increased metabolic and chemical stability^2^. As compared to larger proteins and antibody therapeutics, peptides are small in size, have the ability to penetrate cells and tissues, and their synthesis is more cost-effective^4,5^. Currently, there are over 60 approved peptide drugs on the pharmaceutical market and considerable more in the development pipeline^3^.

An interesting group of peptides for pharmaceutical applications are plant-derived cyclotides. They belong to the large group of RiPPs^2,6^, that are endogenously expressed for instance in the plant families of *Cucurbitaceae*, *Rubiaceae*, *Solanaceae*, *Fabaceae*, *Violaceae*, and *Poaceae*^7^. These peptides are about 30 amino acids in size, and they comprise a head-to-tail cyclized backbone stabilized by three disulfide bonds arranged in a knotted topology. Importantly, these unique structural features render them extremely stable in particular against chemical and enzymatic degradation^8^. Intrinsically, cyclotides exhibit various biological activities, including anti-HIV^9^, immunosuppressive^10^, protease inhibitory^11,12^, uterotonic^13^ and cytotoxicity against cancer cells^14,15^. Besides, cyclotides are amenable to molecular grafting, which allows to introduce an ‘artificial’ peptide sequence into the cyclotide backbone and create a chimeric molecule with novel biological properties^16^. For instance grafted cyclotide molecules can bind to and activate G protein-coupled receptors (GPCRs)^17^, inhibit serine-proteases^18^, inactivate vascular endothelial growth factor^19^, and stimulate angiogenesis^20^. Therefore, cyclotides represent an ideal scaffold for drug discovery with potential for the development as imaging probes or drug lead candidates^21^.

In recent years, numerous studies have unveiled the potential of nature-derived peptides as an extensive source for GPCR ligand design^22,23^. These peptide GPCR ligands have been isolated from various organisms including plants^22^. Accordingly, over 50 peptides targeting GPCRs have been approved as drugs^24^. Cyclotides are a rich source for GPCR ligand discovery, and they have previously been demonstrated to modulate the oxytocin/vasopressin V_1a_ receptors^25^, the corticotropin-releasing factor type 1 receptor^6^, and the κ-opioid receptor^26^. Here, we explored yet another GPCR target of cyclotides namely the cholecystokinin-2 receptor (CCK_2_R). This receptor belongs to an important neuroendocrine system comprising the peptide hormones cholecystokinin (CCK) and gastrin, which mediate their physiological actions through two closely related receptors, i.e. the cholcystokinin-1 receptor (CCK_1_R) and the CCK_2_R (also referred to as CCK_A_R and CCK_B_R)^27,28^. Both receptors are known to be involved in various physiological processes, including the regulation of food intake, increasing pancreatic enzyme secretion and delaying gastric emptying^28^. Importantly, the CCK_2_R has been suggested to participate in tumor development and progression^27,28^. It is often overexpressed in cancer tissue, in particular in gastrointestinal stromal tumors, medullary thyroid cancers, small cell lung carcinomas and insulinomas tumors^29^. Therefore, it is not surprising that the CCK_2_R has been explored as possible drug target for cancer treatment, since reducing the intrinsic activity or blocking of the receptor has yielded promising results in human studies^30^. In this study, we (i) screened three cyclotide-enriched plant extracts of *Oldenlandia affinis*, *Viola tricolor*, and *Carapichea ipecacuanha* for modulation of CCK_2_R signaling, (ii) isolated a particular cyclotide (caripe 11) from *C. ipecacuanha*^6^ and (iii) characterized its pharmacodynamic properties in HEK293 cells overexpressing the CCK_2_R.

## Materials and methods

### Plant material

Plant specimen of *C. ipecacuanha* (Brot.) L.Andersson and *V. tricolor* L. were purchased as powdered material from Alfred Galke GmbH (Germany; catalogue no. 66804 and 13804, respectively). *O. affinis* DC. was grown from seeds (derived from glasshouse grown plants) obtained as a gift from D. Craik (Australia)^25^.

### Peptide extraction and purification

Dried and powdered *C. ipecacuanha*, *O. affinis*, and *V. tricolor* were extracted using dichloromethane:methanol (1:1, v/v) in the ratio of 1:10 (w/v) under permanent stirring at 25 °C for 18–24 h. After filtration, 0.5 volume of ddH_2_O was added to the extract and the aqueous methanol phase was separated by liquid–liquid extraction in a separation funnel. The lyophilized extract was dissolved in ddH_2_O, and peptides were pre-purified in batch using C_18_ silica resin (40–60 µm, ZEOprep 60, ZEOCHEM, Switzerland) activated and equilibrated with methanol and solvent A (0.1% trifluoroacetic acid, TFA in ddH_2_O, v/v), respectively. The resin was washed with 30% solvent B (90% of acetonitrile, ACN, 9.9% of ddH_2_O and 0.1% of TFA, v/v/v) and eluted with 80% solvent B. The eluate was lyophilized and stored at -20 °C until further use. Native cyclotide (caripe 11) was purified by reversed-phase high performance liquid chromatography (RP-HPLC) as previously described^6,25,26^. The purity was assessed by analytical RP-HPLC column (250 × 4.6 mm, 5 µm, 100 Å; Kromasil) at a flow rate of 1 mL/min with a gradient of 5–65% solvent B and by using matrix-assisted laser desorption ionization time-of-flight mass spectrometry (MALDI-TOF MS) as previously described^6,25,26^.

### Peptide quantification

Peptide quantification was carried out by measuring absorbance at 280 nm using a nanodrop instrument and using Beer-Lambert equation. The molar extinction coefficient (Ɛ) for each peptide was determined according to the equation: Ɛ_280_ = nC*120 + nW*5690 + nY*1280 [M^−1^ cm^−1^], where n is the number of residues.

### Cloning of a CCK_2_R-encoding gene and expression vector preparation

A tagged ORF clone encoding the mouse CCK_2_R was purchased from the OriGene (CAT#: MR222564; Germany). The restriction sites of Nhe1 and Xho1 endonucleases were introduced into the CCK_2_R cDNA and amplified using the forward primer: 5′-aaaaaagctagcATGGATCTGCTCAAGCTGAACCG-3′ and the reverse primer: 5′-aaaaaactcgagGCCAGGCCCCAGCGT-3′ (restriction sites are underlined; receptor specific sequence in capital letters). The amplified and digested PCR product was cloned into the pEGFP-N1 plasmid and transfected into competent *E. coli* XL1 cells. Following selection of positive-transfected bacteria, the plasmid was prepared and extracted using the NucleoBond Midi kit (Macherey–Nagel, Germany), quantified using a nanodrop protocol and its sequence confirmed by DNA sequencing. This plasmid produced a receptor with a C-terminal GFP tag; adding a stop codon to the reverse primer, yielded an untagged receptor, which was used for control studies (data not shown).

### Cell culture and transfection

Human embryonic kidney (HEK293) cells (Ref.^31^) were maintained in a fresh Dulbecco’s modified Eagle’s medium supplemented with 10% fetal bovine serum, 100 U/mL penicillin, and 100 µg/mL streptomycin in a humidified atmosphere of 5% CO_2_ at 37 °C. 2 mL of cell suspension was poured into each well of a 6-well plate and incubated overnight at 37 °C. After reaching to a confluency of 70–80%, the cells were transfected with a plasmid encoding EGFP-tagged CCK_2_R using the jetPRIME transfection reagent according to manufacturer’s instructions (Polyplus-transfection, USA).

### Cell viability assays

Effect on cell viability of plant extract was measured against the HEK293 cell line using the Cell Counting Kit-8 (VitaScientific, USA). Briefly, 100 µL of medium containing 1 × 10^4^ cells was seeded in each well of a 96-well plate and incubated at 37 °C for 24 h. Afterwards, 10 µL of various concentration of extract (0–300 µg/mL) or caripe 11 (0.3, 1, 3, 10, 30, 100 µM) was added into each well and then incubated at 37 °C for 2 h. Finally, 10 µL of the kit reagent (final concentration of 10%, v/v) was added into each well and incubated at 37 °C for 3 h. Absorbance was measured at 450 nm using the FlexStation 3 multi-mode microplate reader (Molecular Devices, USA). Triton X-100 and medium were used as positive and negative controls, respectively. The cell viability (CV) percentage was calculated using the following equation: CV (%) = (A_S_/A_C_) × 100, whereas A_S_ and A_C_ are related to the absorbance of sample and negative control at 450 nm.

### Inositol-1-phosphate (IP1) accumulation assay

The receptor-mediated activation of Gq-dependent signaling was measured using a homogenous time-resolved fluorescence (HTRF) based inositol-1-phosphate (IP1) assay kit (Cisbio, France). Approximately 3-6 × 10^4^ HEK293 cells expressing the CCK_2_R were seeded into each well of a 96- or 384-well plate and incubated overnight at 37 °C. Afterwards, the medium was removed, and the cells were stimulated with ‘stimulation buffer’ at 37 °C for 15–30 min followed by incubation with various concentrations of extracts [100 and 300 µg/mL for Caripe (*C. ipecacuanha* extract), 100 µg/mL for Oaff (*O. affinis* extract), and 300 µg/mL for Vitri (*V. tricolor* extract)] and caripe 11 (3, 10 and 30 µM), alone or in combination with 70 nM (EC_80_) of CCK-8 endogenous agonist. Antagonists YM-022 and LY225910 (500 nM or 1 µM, both Sigma -Aldrich, Austria) were preincubated at 37 °C for 30 min. After adding IP1-d2 conjugate and anti-IP1 Eu Cryptate according to manufacturers’ instructions, the plate was incubated at room temperature for 3 h. The HTRF measurement was carried out using the FlexStation 3 multi-mode microplate reader with excitation at 330 nm and emissions at 620 and 665 nm.

### Data analysis

All experiments were performed in triplicate, analyzed using the GraphPad prism software (GraphPad Software, USA) and expressed as mean ± SD (standard deviation). Pharmacological data of concentration response curves were normalized to the maximal response of CCK-8 detected at the highest concentration. The potency (EC_50_) and maximum efficacy (E_max_) were calculated from concentration response curves fitted to the three-parameter non-linear regression with a top and bottom constrained to 100% (for CCK-8) and 0, respectively. Single concentration efficacy data were normalized to baseline (assay buffer) and data were expressed as IP1 formation (fold difference over baseline).

### Ethics statement

The study was carried out in accordance with relevant guidelines and regulation.

## Results

### Preparation and analysis of cyclotide-containing extracts

**Figure 1:**
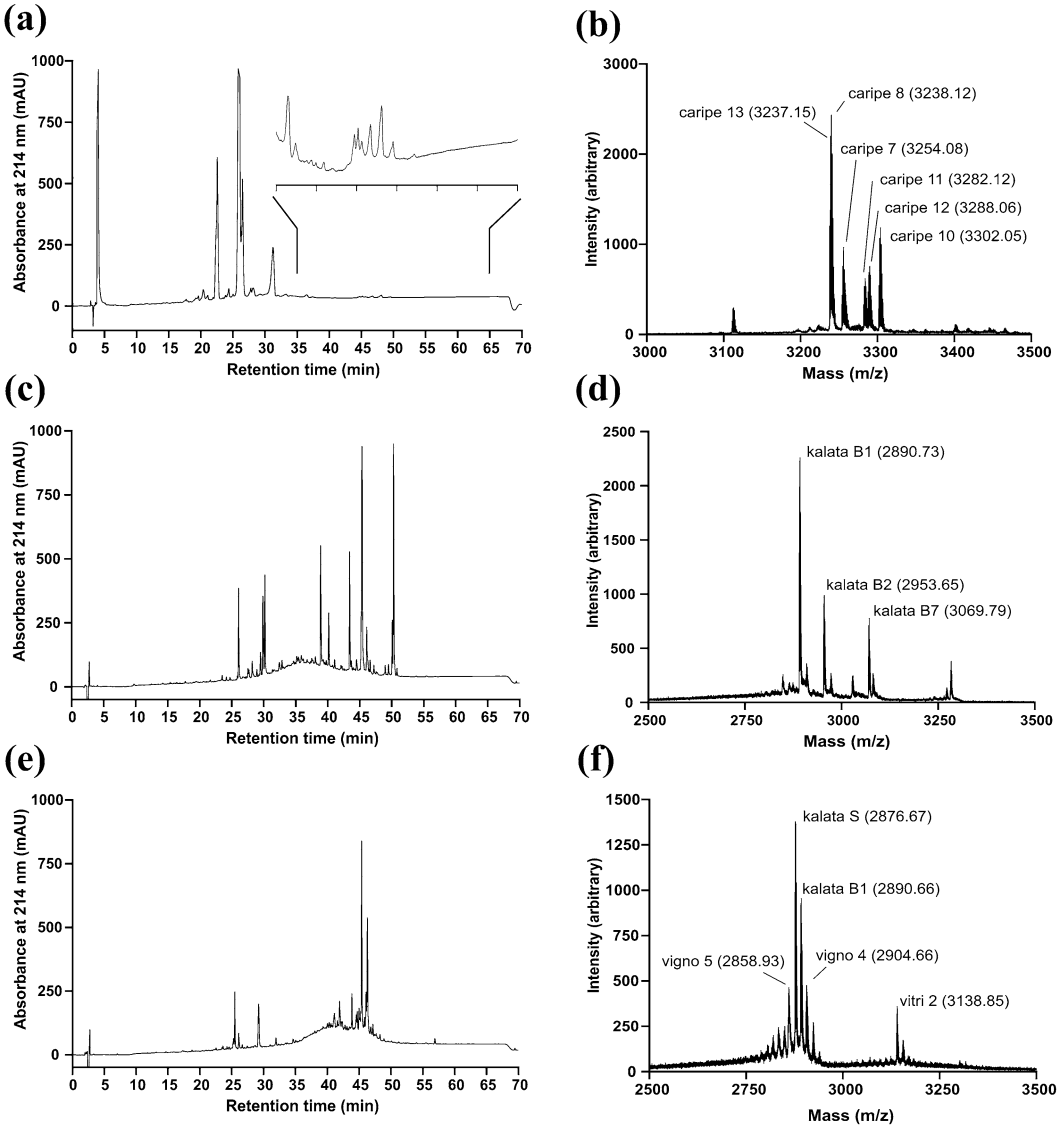
Analytical profile of cyclotide-containing plant extracts. Analytical RP-HPLC chromatograms of the extracts of *C. ipecacuanha* (**a**) with magnification of the cyclotide eluting region (inset), *O. affinis* (**c**), and *V. tricolor* (**e**). MALDI-TOF MS of the extracts of *C. ipecacuanha* (**b**), *O. affinis* (**d**), and *V. tricolor* (**f**), including labelling of identified major cyclotides according to their monoisotopic [M + H]^+^ ions (in parentheses) and common name (according to the CyBase^55^).

For this study we chose three representative cyclotide producing plants, namely *O. affinis* (Oaff), *V. tricolor* (Vitri) and *C. ipecacuanha* (Caripe). The cyclotides elute late in RP chromatography due to their hydrophobic surface properties and a molecular mass between *m/z* 2700–3500 in MALDI-TOF MS^6^. The initial aqueous extracts of Oaff, Vitri and Caripe were prepared by maceration and pre-purified with C_18_ solid-phase extraction to remove polar compounds. Afterward, the presence of cyclotides in these extracts was confirmed and analyzed using RP-HPLC and MALDI-TOF MS (Fig. 1), Cyclotides were assigned by molecular weight according to previous studies^6,31–33^. Accordingly, the major cyclotides (based on their relative peak intensity in MALDI-TOF MS) determined in the Caripe extract were caripe 13 (monoisotopic [M + H]^+^: 3237.15 Da), caripe 8 (3238.12 Da), caripe 7 (3254.08 Da), caripe 11 (3282.12 Da), caripe 12 (3288.06 Da), and caripe 10 (3302.05 Da); in the Oaff extract there were kalata B1 (2890.73 Da), kalata B2 (2953.65 Da), and kalata B7 (3069.79 Da) and in the Vitri extract there were vigno 5 (2858.93 Da), kalata S (2876.67 Da), kalata B1 (2890.66 Da), vigno 4 (2904.66 Da) and vitri 2 (3138.85 Da), respectively (Table 1). Next, we opted to determine the GPCR-modulating activity of cyclotide extracts at the CCK_2_ receptor. Since the extracts still contain many peptides and ‘contaminations’ of small molecules, we first examined suitable concentrations to be used for the pharmacological experiments by performing cell viability assays.

**Table 1:** Sequence alignment and molecular weight of cyclotides analyzed in plant extracts from *O. affinis*, *V. tricolor*, and *C. ipecacuanha.*

Cyclotide	Amino acid sequence	Molecular weight (Da)*
kalata B7	GLPV**C**GET**C**TLGT**C**Y---TQG**C**T**C**SWPI**C**KRN	3069.27
kalata B2	GLPV**C**GET**C**FGGT**C**N---TPG**C**S**C**TWPI**C**TRD	2953.14
vigno 5	GLPL**C**GET**C**VGGT**C**N---TPG**C**S**C**GWPV**C**VRN	2858.15
vigno 4	GLPL**C**GET**C**VGGT**C**N---TPA**C**S**C**SWPV**C**TRN	2904.16
kalata B1	GLPV**C**GET**C**VGGT**C**N---TPG**C**T**C**SWPV**C**TRN	2890.14
kalata S	GLPV**C**GET**C**VGGT**C**N---TPG**C**S**C**SWPV**C**TRN	2876.13
caripe 7	G-IP**C**GES**C**VFIP**C**TVTALLG**C**S**C**KNKV**C**YRN	3253.47
vitri 2	GSIP**C**GES**C**VWIP**C**I-SGIAG**C**S**C**SNKV**C**YLN	3138.32
caripe 13	G-IP**C**GES**C**VFIP**C**F-TSVFG**C**S**C**KDKV**C**YRN	3237.37
caripe 8	GVIP**C**GES**C**VFIP**C**I-TAAIG**C**S**C**KKKV**C**YRN	3237.51
caripe 11	GVIP**C**GES**C**VFIP**C**I-STVIG**C**S**C**KKKV**C**YRN	3281.53
caripe 10	GVIP**C**GES**C**VFIP**C**F-STVIG**C**S**C**KNKV**C**YRN	3301.47
caripe 12	GVIP**C**GES**C**VFIP**C**F-SSVIG**C**S**C**KNKV**C**YRN	3287.45

*Molecular weight is provided as monoisotopic mass taken from CyBase^55^.

### Cell viability assays of cyclotide extracts

**Figure 2:**
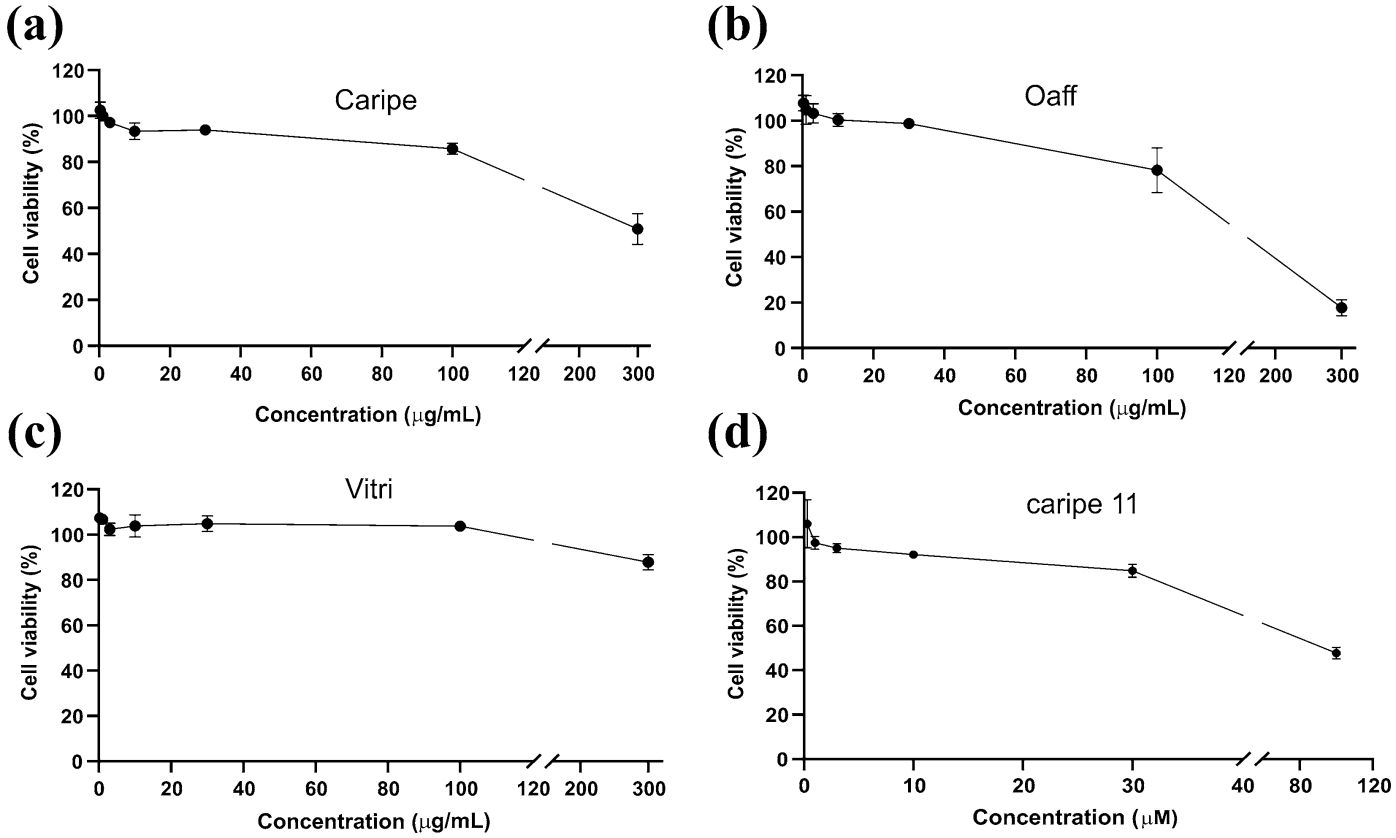
Cytotoxicity of plant extracts and purified cyclotides. Viability of HEK293 cells was determined using the cell counting kit-8 after 2 h incubation with varying concentrations (as indicated in the graphs) of (**a**) extracts of *C. ipecacuanha* (Caripe), (**b**) *O. affinis* (Oaff), (**c**) *V. tricolor* (Vitri), as well as (**d**) purified caripe 11. Cell viability was calculated as percentage using an equation described in the Materials and Methods section using absorbance at 450 nm. Data represent technical triplicates (n = 2) and were expressed as mean ± SD.

The viability of HEK cells to be used for the IP1 second messenger accumulation experiments was determined with extracts of Caripe, Oaff and Vitri. Accordingly, Caripe had no major influence on viability against HEK293 cells up to 100 µg/mL with > 65% of cells remaining viable at 300 µg/mL (Fig. 2a). In addition, the Oaff extract showed no effect at concentrations of < 100 µg/mL, while increasing concentrations of up to 300 μg/mL led to a decrease in viability after 2 h of incubation (Fig. 2b). The Vitri extract exhibited no major effects on viability up to concentrations of 300 µg/mL (Fig. 2c). Therefore, concentrations of up to 300 µg/mL of Caripe and Vitri extracts, and 100 µg/mL of Oaff extract were chosen for further analysis in the IP1 assay.

### Modulating effects of cyclotide plant extracts at CCK_2_R

**Figure 3:**
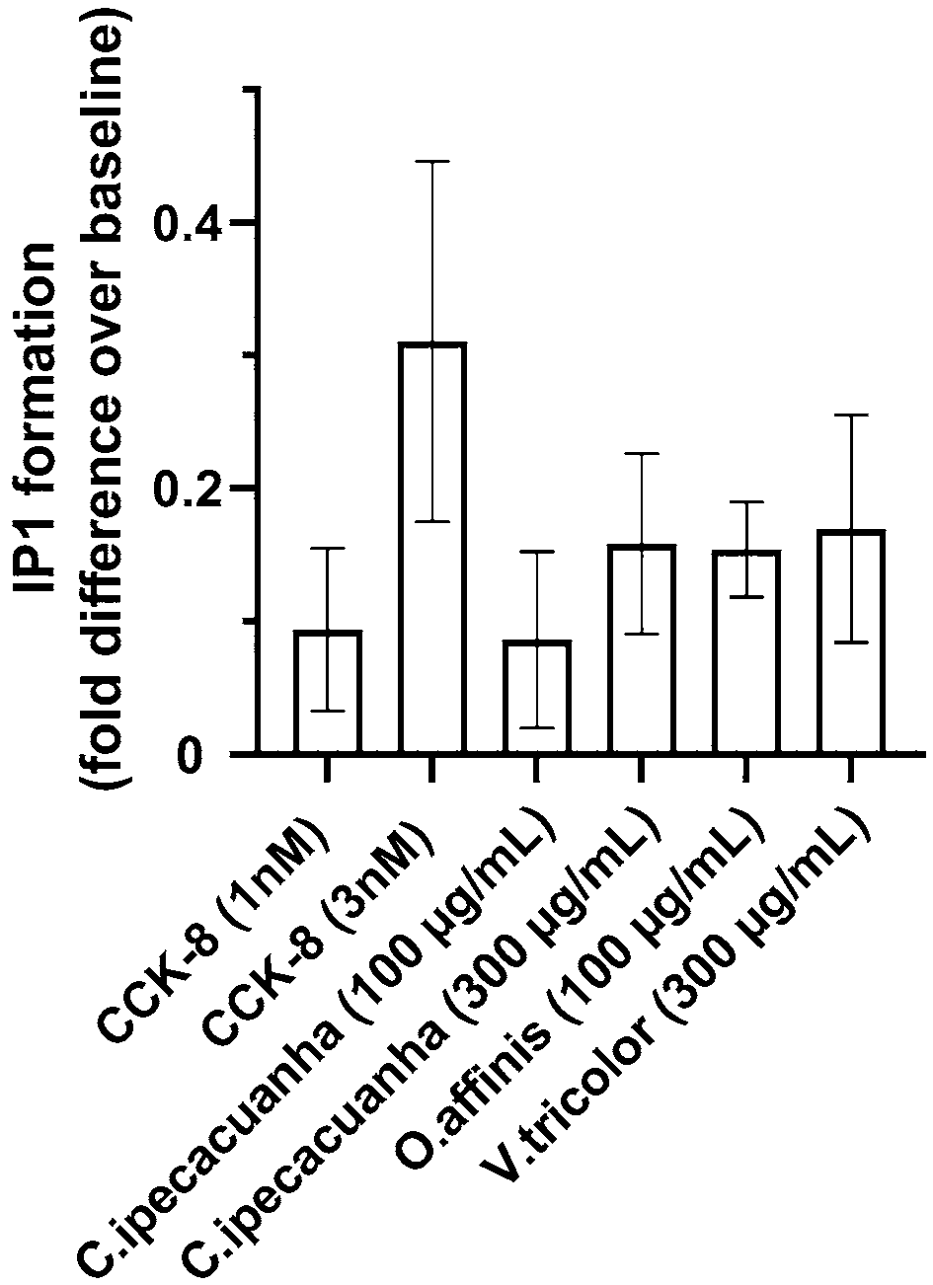
Pharmacological activity of the plant extracts at the CCK_2_R. Agonist efficacy of the plant extracts of *C. ipecacuanha* (Caripe), *O. affinis* (Oaff), and *V. tricolor* (Vitri) were measured by IP1 quantification (fold difference over baseline). Receptor activation was presented as ‘fold difference over baseline’, and data were expressed as mean ± SD (n = 4–5, except Caripe 300 µg/mL is n = 2). Stimulation buffer (not shown) and CCK-8 peptide (1 and 3 nM, n = 3–4) were used as negative and positive controls, respectively.

Cyclotide-containing extracts of Caripe, Oaff and Vitri were screened for their pharmacological activity at the CCK_2_R to detect agonism and antagonism using a commercially available IP1 second messenger assay and HEK293 cells transiently expressing EGFP-tagged CCK_2_R. The pharmacological effect of Caripe extract was determined with two concentrations of 100 and 300 µg/mL, respectively, whereas one concentration was used for the Oaff (100 µg/mL) and Vitri (300 µg/mL) extracts. The functionality of the receptor was validated by CCK-8, the endogenous CCK_2_R peptide ligand. As expected, CCK-8 produced a concentration-dependent increase in IP1 (Fig. 3), an effect that was absent in non-transfected HEK293 cells (data not shown). All three plant extracts produced a moderate accumulation of IP1 (Fig. 3): Caripe at 300 µg/mL (0.16 fold difference over baseline), Oaff at 100 µg/mL (0.15) and Vitri at 300 µg/mL (0.17) had a comparable efficacy at IP production as the endogenous agonist CCK-8 at 1 and 3 nM (0.09 and 0.31), respectively. These results indicate the presence of molecules in these extracts that are capable of activating the CCK_2_R. Given its ethnopharmacological importance as former phytomedicine, a representative extract of Caripe (also known as the ‘syrup of ipecac’^34^) was chosen for isolation of a purified cyclotide and further analysis to determine the pharmacological effect of a cyclotide at the CCK_2_R.

### Purification of caripe 11

**Figure 4:**
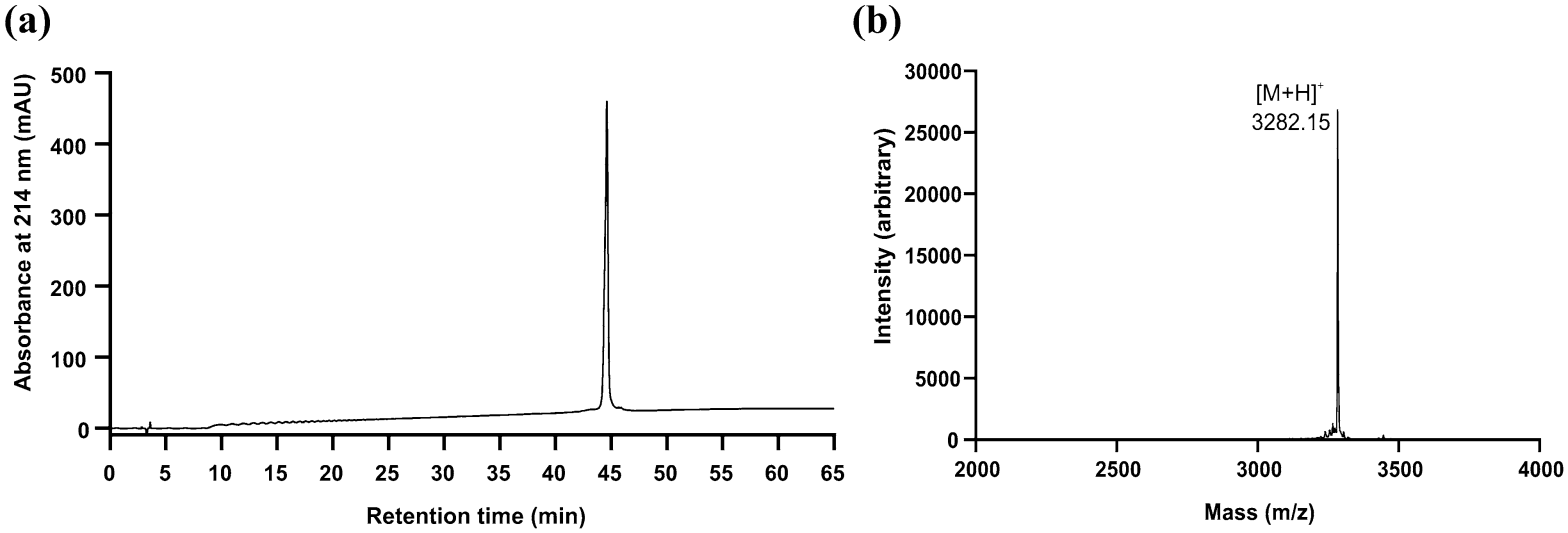
Analytical profile of native caripe 11 cyclotide isolated from *C. ipecacuanha*. The quality control using RP-HPLC (**a**) and MALDI TOF MS (**b**) with yielded 99.8% purity, whereas labeled m/z refers to monoisotopic [M + H]^+^ ion.

Previously, we have demonstrated the pharmacology-guided isolation of cyclotides from *C. ipecacuanha* and other cyclotide-containing plants^6,33^. *C. ipecacuanha* is known to contain several cyclotides with closely related sequences (Table 1)^6,33^. Therefore, we isolated a representative cyclotide, namely caripe 11, to determine its properties to modulate CCK_2_R signaling. The cyclotide was purified using preparative RP-HPLC^6^ and its purity and identity were determined using analytical RP-HPLC and MALDI-TOF MS, respectively, which yielded the cyclotide caripe 11 with 99.8% purity and a molecular weight of 3281.3 Da (Fig. 4). For cell-based assays, the concentration of a caripe 11 solution was calculated using the absorbance at 280 nm (extinction coefficient: 2000 M^−1^ cm^−1^). Furthermore, we determined its effect on cell viability; concentrations of ≤ 30 µM of caripe 11 did not have a pronounced effect, but the concentration of 100 µM slightly decreased HEK cell viability (Fig. 2d). Therefore, concentrations of up to 30 µM of caripe 11 were considered as appropriate for further analysis.

### Caripe 11 is a partial agonist of the CCK_2_R

**Figure 5:**
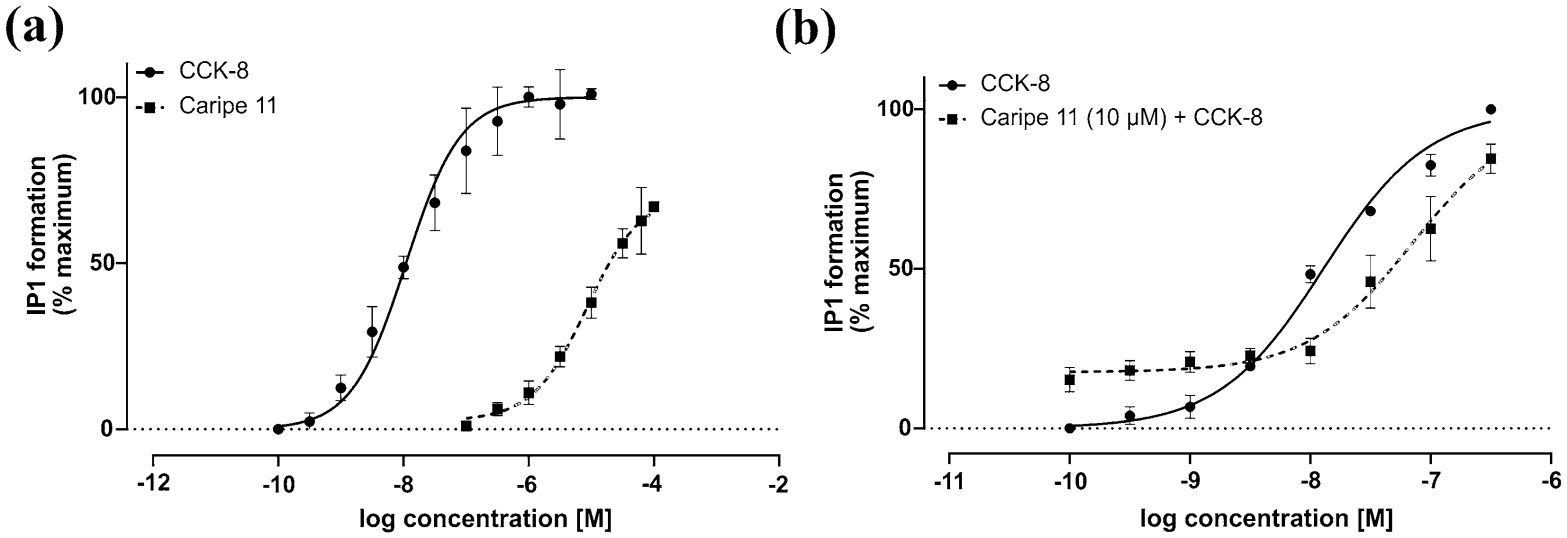
Partial agonist activity of caripe 11 at the CCK_2_R. (**a**) Concentration–response curves of the effect of CCK-8 and caripe 11 upon activation of the receptor via measurement of the intracellular formation of IP1. Potency and efficacy values are listed in Table 2. (**b**) Concentration–response curves of CCK-8 alone and in combination with caripe 11 (10 µM). Stimulation buffer and CCK-8 peptide were used as negative and positive controls with 0 and 100% efficacy, respectively. Each experiment was carried out in technical triplicate and three (n = 3) biological repeats. All data are shown as mean ± SD.

Partial agonists of GPCRs are defined as ligands that trigger submaximal efficacy at the receptor as compared to full agonists^35^. To determine the activity of caripe 11 we measured concentration–response curves of the CCK-8 control agonist and caripe 11 using HEK293 cells transiently expressing CCK_2_R. By definition, CCK-8 is an endogenous peptide ligand that per se fully activates the receptor. Our results demonstrate that CCK-8 activates the receptor with a potency (EC_50_) of 11.5 nM. On the other hand, caripe 11 activates the receptor with a submaximal efficacy (E_max_ = 71%) and a potency of 8.5 µM compared to CCK-8 (Fig. 5a and Table 2). This suggests that caripe 11 is a partial agonist of the CCK_2_R with moderate potency. To confirm the pharmacological mechanism of caripe 11, we determined the effects of three concentrations of caripe 11 upon pre-treatment of cells with an EC_80_ concentration of CCK-8. As expected, caripe 11 concentration-dependently decreased the EC_80_ effect of CCK-8 when both ligands were co-incubated. Accordingly, caripe 11 (30 µM) was able to reduce the EC_80_ effect (70 nM) of CCK-8 by ~ 20% (Fig. S1a). Furthermore, in the concentration–response curve the basal activity of CCK-8 was increased (0 to 17%) upon co-treatment with caripe 11 (10 µM) (Fig. 5b), a phenomenon commonly observed for partial agonists^36^. Moreover, the curve of CCK-8 in combination with caripe 11 was shifted to the right; this led to a nearly sixfold decrease in the potency of CCK-8 from an EC_50 CCK-8_ of 12.9 nM to an EC_50 CCK-8+caripe11_ of 71 nM (Fig. 5b). These characteristics suggest that caripe 11 is a partial agonist of the CCK_2_R.

**Table 2:** Potency (EC_50_) and maximum efficacy (E_max_) of CCK-8 and caripe 11 at the CCK_2_R.

CCK-8			caripe 11		
EC_50_	logEC_50_	E_max_	EC_50_	logEC_50_	E_max_
11.5 ± 1.5 nM	− 7.9 ± 0.06	100	8.5 ± 1.7 µM	−5.1 ± 0.08	71 ± 6.9

Data are shown as mean ± SD based of three biological measurements (n = 3).

## Discussion

Cyclotides are cyclic plant peptides comprising a unique structural topology that is currently being explored in drug discovery and development. Cyclotides and many other nature-derived peptides occupy a chemical space that is different from small molecules, and therefore they may be able to interact with proteins otherwise difficult to target by small molecules. The use of plants in traditional medicine for the discovery of new pharmaceuticals and lead compounds is one of the central dogmas of ethnopharmacology and pharmacognosy^1,25^. The prototypical cyclotide plant *O. affinis*, known for its traditional use in childbirth and post-partum care, was the source for the first nature-derived cyclotide/GPCR ligand, kalata B7, which acts as a partial agonist at the oxytocin- and vasopressin V_1a_-receptors^25^. Meanwhile a number of different cyclotide GPCR ligands has been discovered and synthesized^23^, and hence in this study we aimed at increasing the repertoire of GPCR-modulating cyclotides by exploring the CCK_2_R.

GPCRs are one of the largest group of membrane proteins in the human body with over 800 unique receptor sequences known to date^37^. The CCK_2_R is a class A GPCR that is relevant in many physiological and pathological processes, e.g. it is involved in several metabolic and gastric disorders^30^, as well as cancer^29^. Based on the therapeutic potential of CCK_2_R and therefore the need for new ligands targeting this receptor, in this study we screened cyclotide-containing extracts of *O. affinis*, *C. ipecacuanha*, and *V. tricolor* to explore their ability to modulate CCK_2_R signaling. The content and nature of cyclotides in these extracts were analyzed and confirmed by RP-HPLC and MALDI-TOF MS. In line with the defining criteria of cyclotides, peptides identified in the three plant extracts were late-eluting in RP-HPLC and exhibited a molecular mass between 2700 and 3500 Da^38^. We pharmacologically screened these cyclotides-enriched extracts in a functional IP1 assay using HEK293 cells overexpressing the mouse CCK_2_R. All three tested extracts exhibited the ability to modulate Gq-dependent CCK_2_R signaling. To demonstrate specificity of these effects we tried co-treatment with two competitive human CCK_2_R inhibitors, YM-022^39^ and LY225910^40^. Unfortunately, it was not possible to block the agonist effects of the extracts (data not shown). Reasons for this are discussed in the following: (i) the antagonists were specifically designed to the human receptor; however, in our study we used the mouse receptor. Not surprisingly, LY225910 did not displace the effects of endogenous ligand CCK-8; to demonstrate an antagonistic effect of YM-022 we had to use high concentrations (> 500 nM) despite its reported picomolar affinity (Fig. S1b). (ii) Some compounds in the extract (which contain up to hundreds of peptides and other molecules)^38^ may interfere with the activity of the antagonist, and (iii) cyclotides may form a stable complex with the receptor that cannot easily be displaced by the antagonist; this phenomenon has been observed for other GPCRs previously (summarized in Ref.^41^). For instance, partial agonists of the adenosine receptor such as LUF7746 bind covalently and cannot be displaced by antagonists^42,43^, and the same is true for the cannabinoid 1 receptor ligand AM841^44^.

To identify cyclotides responsible for the modulation of CCK_2_R signaling, we next isolated several cyclotides from a representative cyclotide extract of *C. ipecacuanha*. At least one of these peptides, namely caripe 11 exhibited the ability to partially activate CCK_2_R. This partial agonism of caripe 11 was further analyzed by co-incubation of the cyclotide with CCK-8 and caripe 11, which led to a dextral shift of the CCK-8 concentration–response curve (i.e., decrease of CCK-8 potency). These findings are in line with our previous studies that identified kalata B7 cyclotide to be a partial agonist of oxytocin and vasopressin receptors^25^. Furthermore, caripe cyclotides, first reported by Koehbach et al*.*^33^, have been demonstrated to function as antagonists at the corticotropin-releasing factor type 1 receptor^6^ and agonists of the κ-opioid receptor^26^.

Because partial agonists of GPCRs trigger submaximal effector coupling and thus induce less receptor desensitization as compared to full agonists, they provide opportunities to develop pharmacotherapies with improved side effect profiles^45^. A prime example is salmeterol, a partial agonist of β_2_-adrenoceptor^46^ in clinical use for treatment of asthma and chronic obstructive pulmonary disease^47^. Furthermore, buprenorphine displays partial agonist activity at the µ-opioid receptor yet exerts pharmacological effects similar to an antagonist^48^. In fact, these characteristics of buprenorphine make it an attractive compound for clinical use in pain management^49^ and opioid dependence^50^.

The unique features of cyclotides have led to their use in the design of cyclotide-based drugs with improved pharmacological properties. Due to their capability to accommodate structural variations, cyclotides have been extensively utilized as molecular scaffolds to design new molecules and ligands with interesting biological features by applying ‘molecular grafting’^51^. For instance, Camarero et al*.* designed and synthesized a cyclotide-based antagonist of the HIV-1 viral replication that is able to target the chemokine receptor CXCR4^52^. In addition, the grafted cyclotide MCo-CVX-5c was used as a template for designing and synthesizing MCo-CVX-6D (the Lys residue in loop 1 has been conjugated to DOTA) that exhibited affinity at CXCR4 in the sub-nanomolar range^53^. These studies demonstrated feasibility for grafting GPCR-binding peptide motifs into the cyclotide framework. The grafted CXCR4 cyclotides are important and stable cancer imaging tools^53^. Accordingly, the affinity of the cyclotide caripe 11, identified in this current study, may be exploited as a scaffold for molecular grafting to design CCK_2_R ligands with improved pharmacological properties, e.g., enhanced potency/affinity and stability. Given the therapeutic potential of CCK_2_R in the treatment of cancer^27,29^, for instance, by grafting of the endogenous CCK-8 sequence into the cyclotide scaffold, and the conjugation of an imaging reagent could yield a high-affinity cancer imaging probe. In this study, we did not determine activity of caripe 11 at the related CCK_1_R, and therefore we cannot address receptor-subtype selectivity. Proof-of-principle for such a grafting application, and possibly to investigate receptor selectivity will have to be provided in future studies.

At a more general level, our work provides yet another example that cyclotides are capable of modulating GPCR signaling^23^. Their functional diversity, structural plasticity and high stability make them suitable scaffolds to develop new GPCR-targeting ligands with unique pharmacological properties^23^. In fact, cyclotides are amenable to molecular grafting, which facilitates the engineering of chemical probes and ligands of GPCRs^23,53,54^. Here, we discovered for the first time a cyclotide ligand of the CCK_2_R that may for instance be utilized as a stable labelled ligand in imaging applications, as a gut-stable probe, or as a scaffold for designing stabilized peptide ligands of the CCK_2_R. Thus, our study helped to increase the rich diversity of cyclotides as ligands of GPCR and points to their potential use as starting points for the design of cyclotide-based ligands targeting CCK_2_R to treat human illnesses such as cancer.

## Conclusion

GPCRs remain privileged drug targets. The CCK_2_R is an example of a GPCR with therapeutic potential for the treatment of gastrointestinal disorders including cancer. Cyclotides are nature-derived peptides that represent an emerging class of GPCR modulators. In this study, we demonstrated a cyclotide that modulates CCK_2_R signaling as a partial agonist. Therefore cyclotides may be utilized as templates for designing new GPCR ligands with unique pharmacological properties^25^.

## Supplementary Information

Supplementary Figure S1.
