# Supplementary material for: Discovery of the cyclotide caripe 11 as a ligand of the cholecystokinin-2 receptor
Source: Sci Rep. 2022 Jun 2;12:9215. doi: 10.1038/s41598-022-13142-z (PMC9163038; doi:10.1038/s41598-022-13142-z)
Supplement: Supplementary file 1 — Supplementary Figure S1. [file 41598_2022_13142_MOESM1_ESM.docx]

**Supporting Data**

**Discovery of the cyclotide caripe 11 as a ligand of the cholecystokinin-2 receptor**

Mohammad Sadegh Taghizadeh^1,2^, Bernhard Retzl^1^, Edin Muratspahić^1^, Christoph Trenk^1^, Emilio Casanova^1^, Ali Moghadam^2^, Alireza Afsharifar^3^, Ali Niazi^2^, Christian W. Gruber^1^

^1^Center for Physiology and Pharmacology, Medical University of Vienna, 1090 Vienna, Austria

^2^Institute of Biotechnology, Shiraz University, Shiraz, Iran

^3^Center of Plant Virology Research, Shiraz University, Shiraz, Iran

**
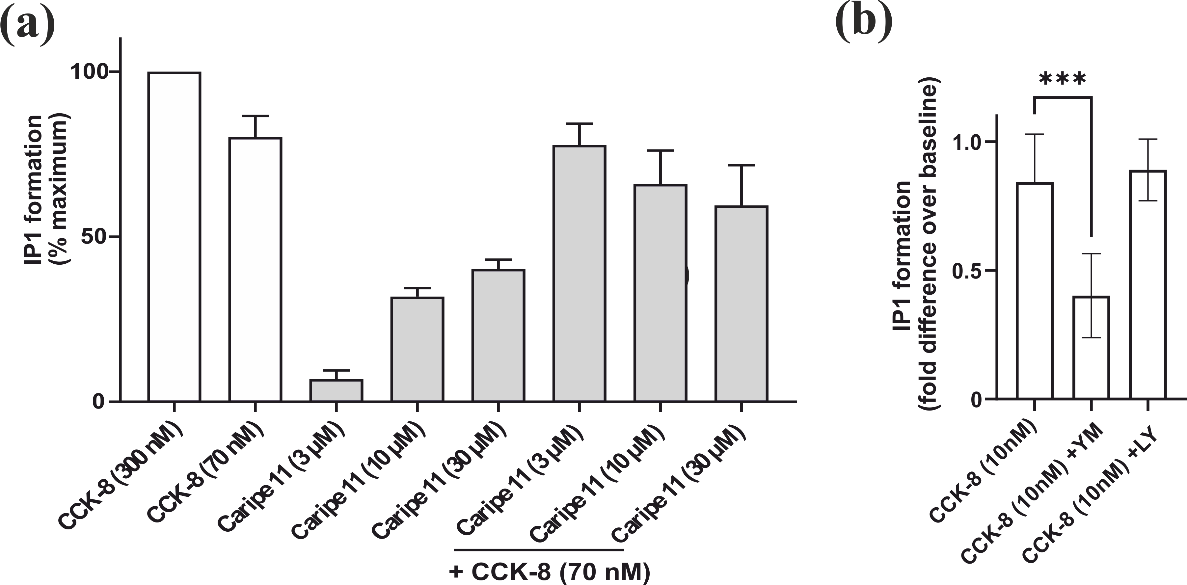
**

**Figure S1.** Pharmacological control experiments at the CCK_2_R. **(a)** Activity of caripe 11 (3, 10 and 30 µM) in the presence of an EC_80_ concentration of the endogenous full agonist CCK-8 (70 nM). **(b)** The specificity of CCK-8 (10 nM) was determined by co-treatment with two antagonists (YM-022, 500 nM; LY225910, 500 nM). Only one antagonist (YM-022) produced a significant inhibition of the IP1 formation (shown as fold difference over baseline) (*p<0.001*). Each experiment was carried out in technical triplicate, with two (a) (n = 2), or (b) at least three (n=3-5) biological repeats, respectively. All data are shown as mean ± SD.
